# Water Extract of *Mentha arvensis* L. Attenuates Estrogen Deficiency-Induced Bone Loss by Inhibiting Osteoclast Differentiation

**DOI:** 10.3389/fphar.2021.719602

**Published:** 2021-08-05

**Authors:** Seon-A Jang, Youn-Hwan Hwang, Hyun Yang, Jin Ah Ryuk, Taesoo Kim, Hyunil Ha

**Affiliations:** KM Convergence Research Division, Korea Institute of Oriental Medicine, Daejeon, South Korea

**Keywords:** mentha arvensis, osteoporosis, osteoclastogenesis, ovariectomy, RANKL

## Abstract

*Mentha arvensis* L., is an aromatic herb that belongs to the Lamiaceae family and is widely used in medicinal applications, essential oil applications, and food flavoring. The extract of *M. arvensis* has been reported to exert sedative-hypnotic, anti-inflammatory, anti-fungal, and anti-bacterial effects. However, its effects on bone metabolism have not yet been studied. Here, we investigated the effects of the water extract of *M. arvensis* (WEMA) on osteoclast formation *in vitro* and bone loss in an ovariectomized mouse model. We found that WEMA inhibited osteoclast differentiation by directly acting on osteoclast precursor cells. WEMA inhibited receptor activator of nuclear factor-κB ligand (RANKL)-induced the expression of cellular oncogene fos (c-Fos) and nuclear factor of activated T cells c1 (NFATc1), crucial transcription factors for osteoclast differentiation, by suppressing RANKL-induced activation of early signaling pathways such as those of mitogen-activated protein kinases (MAPKs) and nuclear factor-κB (NF-κB). In addition, oral administration of WEMA suppressed ovariectomy-induced trabecular bone loss in mice. We additionally identified phytochemicals in WEMA that are known to have anti-osteoclastogenic or anti-osteoporotic properties. Collectively, these results suggest that WEMA is a promising herbal candidate that can be used to prevent or treat postmenopausal osteoporosis.

## Introduction

Bone homeostasis is maintained by a balance between bone resorption and bone formation, mediated by osteoclasts and osteoblasts, respectively. An imbalance in bone homeostasis can occur due to abnormal bone resorption of osteoclasts or loss of osteoblast function. Bone imbalance caused by excessive osteoclast-mediated bone resorption is closely associated with most bone metabolic diseases such as osteoporosis, periodontitis, rheumatoid arthritis, metastatic cancers, and multiple myeloma (Boyle et al., 2003; Tanaka et al., 2005).

Osteoporosis is one of the major health problems in aging communities. There are two types of osteoporosis: type 1 is postmenopausal osteoporosis occurring in postmenopausal women, and type 2 is senile osteoporosis occurring in both women and men over 70 years of age (Boonen et al., 1996). In postmenopausal osteoporosis, loss of trabecular bone is increased, whereas in senile osteoporosis, it increases both the loss of cortical and trabecular bone.

Currently, hormone replacement therapy (HRT) and bisphosphonates are the representative treatments used for postmenopausal osteoporosis. However, long-term HRT has been found to increase the risk of breast cancer, cardiovascular diseases, and biliary disease, and bisphosphonates cause osteonecrosis of the jaw (Nelson et al., 2002; Diel et al., 2007). Therefore, there is a growing interest in the use of natural medicinal plants for therapy that are thought to have few side effects.

Osteoclasts are giant multinucleated cells derived from hematopoietic precursor cells in the bone marrow. Osteoclast precursor cells can differentiate into mature osteoclasts when exposed to the macrophage colony-stimulating factor (M-CSF) and receptor activator of NF-κB ligand (RANKL) (Boyle et al., 2003). RANKL is an essential source of precursor cells that differentiate into osteoclasts, and M-CSF provides the survival of these cells. The binding of RANKL to its receptor promotes the recruitment of tumor necrosis factor receptor-associated factor 6 and activates multiple downstream signaling pathways, including those of nuclear factor-κB (NF-κB) and mitogen-activated protein kinases (MAPKs) including c-Jun N-terminal protein kinase (JNK), p38, and extracellular signal-regulated kinase (ERK). This signaling induces the activation of transcription factors for osteoclastogenesis, namely, c-Fos and nuclear factor of activated T cells c1 (NFATc1), and results in the expression of osteoclast-specific genes such as tartrate-resistant acid phosphatase (TRAP), matrix metalloproteinase-9 (MMP-9), cathepsin K, integrin β3, and calcitonin receptor (Boyle et al., 2003; Wittrant et al., 2003). Inhibition of these molecules could be an effective treatment for osteoporosis by interfering with osteoclast differentiation and bone resorption of activated osteoclasts.

*Mentha arvensis* L., an aromatic herb belonging to the Lamiaceae family, is extensively cultivated in India, Japan, Nepal, Bangladesh, and Srilanka for its use in medicinal applications, essential oil applications, and food flavoring (Thawkar, 2016). Previous studies have shown that *M. arvensis* contains various phytochemicals mainly including monoterpenes (e.g., menthol, menthone, and isomenthone), phenolic acids (e.g., caffeic acid and rosmarinic acid), and flavonoids (e.g., luteolin, linarin, and hesperidin) (Akram et al., 2011). *M. arvensis* has traditionally been used to treat bacillary dysentery, flatulence, dyspepsia, gastritis, and enteritis. Recently, *M. arvensis* has been reported to have various beneficial biological properties including sedative-hypnotic, anti-inflammatory, anti-fungal, and anti-bacterial activities (Akram et al., 2011). However, the pharmacological properties of the water extract of *M. arvensis* (WEMA) on osteoporosis have not been studied. In this study, we investigated the bone protective effects of WEMA on RANKL-induced osteoclastogenesis and ovariectomy (OVX)-induced postmenopausal osteoporosis.

## Materials and Methods

### Materials

Cell Counting Kit-8 (CCK-8) was obtained from Dojindo Molecular Technologies Inc. (Rockville, MD, United States). Alpha-modified minimal essential medium (α-MEM) was purchased from Hyclone (Logan, UT, United States). Fetal bovine serum (FBS) and calf serum were obtained from Thermo Fisher Scientific (Waltham, MA, United States). 1α,25-dihydroxyvitamin D3 (VitD_3_) was purchased from Sigma-Aldrich (St. Louis, MO, United States). Recombinant human M-CSF was obtained from Dr. Yongwon Choi (University of Pennsylvania School of Medicine, Philadelphia, PA, United States). Recombinant soluble GST-tagged human RANKL was prepared as previously described (Ha et al., 2013). Antibodies against phospho-IκBα (Ser32), IκBα, phospho-JNK1/2 (Thr183/Tyr185), JNK, phospho-ERK1/2 (thr202/Tyr204), ERK, phospho-p38 (Thr180/Tyr182), p38, and β-actin were purchased from Cell Signaling Technology (Danvers, MA, United States). Antibody against aryl hydrocarbon receptor (AhR) was purchased from Enzo Life Sciences, Inc. (Farmingdale, NY, United States). Antibodies against c-Fos and NFATc1 were purchased from Santa Cruz Biotechnology (Santa Cruz, CA, United States). WEMA was purchased from the National Development Institute of Korean Medicine (Gyeongsan, Korea). In brief, leaves of *M. arvensis* were extracted with distilled water at reflux for 3 h and dried using a vacuum freeze dryer after filtration. The WEMA powder was re-suspended in distilled water, centrifuged at 10,000 × g for 5 min, and stored at −20°C until required for experiment.

### Bone Marrow-Derived Macrophages (BMMs) Isolation

Bone marrow cells were isolated from 7-week-old male C57BL/6 J mice. The femur and tibia were aseptically extracted, and the marrow cavity was flushed out with α-MEM from one end of the bone using a sterile needle. Single cells obtained from the bone marrow suspension using a cell strainer (70 µm), and red blood cells were lysed for 5 min using Ammonium-Chloride-Potassium (ACK) lysing buffer. The cells were washed twice and incubated with α-MEM in the presence of M-CSF (20 ng/ml) for 24 h. Non-adherent cells were harvested, washed, resuspended in α-MEM in the presence of M-CSF (60 ng/ml), and cultured in non-coated plates for 5 days. Non-adherent cells were removed by washing with PBS and then BMMs were incubated for 10 min with an Enzyme Free Cell Dissociation Solution (EDM Millipore Corp., Burlington, MA, United States), and harvested using a cell lifter.

### Cell Culture

The murine osteocyte-like cell line, MLO-Y4 cells at 40 passages (Kerafast, Boston, MA, United States) were cultured in growth medium consisting of α-MEM supplemented with 2.5% FBS, 2.5% calf serum, and 1% penicillin/streptomycin on type I collagen-coated plates. MLO-Y4 cells were derived from the long bone of a transgenic mouse expressing a T-antigen transgene under the control of the osteocalcin promoter (Bonewald, 1999), and utilized phenotypic criteria of high osteocalcin expression and morphology consistent with osteocytes. BMMs were cultured in α-MEM supplemented with 10% FBS, 1% penicillin/streptomycin, and M-CSF (60 ng/ml).

### Cell Viability

The BMMs (2 × 10^4^ cells/well) were seeded in 96-well plates and after 12 h, the medium was replaced with α-MEM with or without WAMA (11.1, 33.3, 100, and 200 μg/ml). After 24 h, cell viability was assessed by a CCK-8 assay, and absorbance was measured at 450 nm using a conventional microplate reader (Molecular Devices, San Jose, CA, United States).

### Osteoclast Differentiation

For osteoclast differentiation assay in BMM-osteocyte co-culture, MLO-Y4 (1 × 10^3^ cells/well) cells were cultured in a 96-well plate in α-MEM supplemented with 10% FBS and 1% penicillin/streptomycin. The next day, BMMs (4 × 10^4^ cells/well) were added to the culture of MLO-Y4, and the co-cultures were cultured with or without WEMA for 5 days in the presence of VitD3 (10 nM) or VitD3 (10 nM) plus RANKL (50 ng/ml). For osteoclast differentiation in BMMs, BMMs (1 × 10^4^ cells/well) were cultured with or without WEMA for 4 days in the presence of M-CSF (60 ng/ml) and RANKL (50 ng/ml) in a 96-well plate.

### TRAP Activity and Staining

To measure TRAP activity, cells were washed with PBS, fixed using 10% neutral buffered formalin, permeabilized using 0.1% Triton X-100 for, and then incubated using TRAP activity solution (50 mM sodium tartrate, 0.12 M sodium acetate, and p-nitrophenyl phosphate, pH 5.2) for 15 min at 37°C. The reaction was stopped using 0.1N NaOH and the absorbance was measured at 405 nm using a microplate reader. After measuring the TRAP activity, cells were washed with distilled water and stained using TRAP staining solution (50 mM sodium tartrate, 0.12 M sodium acetate, naphthol AS-MX phosphate, and fast red violet LB salt, pH 5.2). Color development was stopped by washing with distilled water. TRAP-positive multinucleated cells (MNCs) containing three or more nuclei were counted as osteoclasts.

### Western Blot Analysis

Whole cell proteins were extracted from BMMs using lysis buffer (iNtRON Biotechnology, Sungnam, Korea). Protein concentrations were determined using a standard curve based on the BSA standard reagent that was included in the BCA Protein Assay Kit (ThermoFisher Scientific, MA, United States). The proteins were separated on a 10% SDS-PAGE gel and transferred to a polyvinylidene fluoride membrane. The membranes were blocked with 5% skim milk, incubated with primary antibodies (1:1,000 dilution) against AhR, c-Fos, NFATc1, p-ERK, ERK, p-JNK, JNK, p-p38, p38, p-IκBα, IκBα, or β-actin overnight at 4°C, and then washed 6 times for 5 min each with Tris-buffered saline with 0.1% Tween 20 (TBST) at room temperature. The membranes were incubated with horseradish peroxidase-conjugated secondary antibodies (1:5,000 dilution) for 1 h at room temperature and washed six times with TBST. Specific bands were detected using SuperSignal^®^ West Pico Chemiluminescent Substrate and visualized using the ChemiDoc Imaging System (Bio-Rad, Hercules, CA, United States).

### Quantitative Real-Time Polymerase Chain Reaction (qPCR)

Total RNA was extracted using the RNA-spin^TM^ Total RNA Extraction Kit (iNtRON Biotechnology, Sungnam, Korea) according to the manufacturer’s instructions. Total RNA (1 μg) was used for cDNA synthesis using a High-Capacity cDNA Reverse Transcription Kit (Thermo Fisher Scientific, Waltham, MA, United States). qPCR was performed on an ABI 7500 Real-Time PCR Instrument (Applied Biosystems) after mixing the cDNA (25 ng), TaqMan Universal Master Mix II (Applied Biosystems, Foster City, CA, United States) and TaqMan probe for NFATc1 (Mm00479445_m1), c-Fos (Mm00487425_m1), B lymphocyte-induced maturation protein 1 (Blimp1, Mm00476128_m1), interferon regulatory factor-8 (IRF-8, Mm00492567_m1), v-maf avian musculoaponeurotic fibrosarcoma oncogene homolog B (MafB, Mm00627481_s1), MMP-9 (Mm00442991_m1), integrin β3 (Mm00443980_m1), cathepsin K (Mm00484036_m1), and 18S (Mm99999915_g1). Fold changes in target gene expression were calculated using the ΔΔCt method.

### *In Vivo* Study

All animal experiments were approved by the Institutional Animal Care and Use Committee (IACUC) at Knotus (Guri, Korea) (approval number: 19-KE-216). Female C57BL/6 J mice (6 weeks old) were housed under standard laboratory conditions of humidity (55% ± 5%), temperature (22°C ± 2°C), and illumination circle (12 h light/dark cycle). The mice were bilaterally ovariectomized or sham operated through dorsal incisions under Zoletil and Rumpun anesthesia. One week after surgery, healthy mice that recovered from OVX surgery were selected and randomly assigned into four groups (*n* = 6): sham group, OVX group, OVX + WEMA 100 mg/kg/day treatment group (WEMA-L), and OVX + WEMA 300 mg/kg/day treatment group (WEMA-H). The mice were provided a commercial normal-fat diet (Research Diet, New Brunswick, NJ, United States) and water ad libitum, and WEMA was administered once daily by oral gavage for 5 weeks.

### Trabecular Bone Analysis

The distal femurs of mice were fixed by 10% neutral buffered formalin for 2 days and then scanned using a µ-computed tomography (µ-CT) imaging system (Bruker, Kontich, Belgium). After scanning the femur, reconstruction and analysis of the original images were performed using SkyScan NRecon and CTAn software, respectively. Trabecular morphometric parameters including bone mineral density (BMD), bone volume per tissue volume (BV/TV), trabecular thickness (Tb.Th), trabecular number (Tb.N), and trabecular separation (Tb.Sp) were calculated.

### Ultrahigh-Performance Liquid Chromatography–Tandem Mass Spectrometry (UHPLC–MS/MS)

A Dionex UltiMate 3,000 system equipped with a Thermo Q-Exactive mass spectrometer was used to analyze the WEMA constituents. Chromatographic separation was achieved using an Acquity BEH C18 column (100 × 2.1mm, 1.7 µm). A gradient elution of the mobile phase using 0.1% formic acid in water (A) and acetonitrile (B) was set as follows: 0–1 min, 3% B; 1–2 min, 3–15% B; 2–13 min, 15–50% B; 13–20 min, 50–100% B; 20–23 min, 100% B; and 23.5–27.5 min, 3% B. The Q-Exactive mass spectrometer was equipped with a heated electrospray ionization source and operated in the positive and negative ion switching modes, according to a previous study (Hwang et al., 2019). Data acquisition and analysis were performed using Xcalibur v.3.0 and Tracefinder v.3.2 software. Neochlorogenic acid was obtained from ChemFace (Wuhan, China). Danshensu, chlorogenic acid, rutin, isoquercitrin, ferulic acid, hesperidin, rosmarinic acid, and linarin were obtained from Targetmol (Wellesley Hills, MA, United States). The identification of phytochemicals from WEMA was performed by comparing the retention time and mass spectral pattern with reference standards or according to a previous report (Hanafy et al., 2017).

### Statistical Analysis

Data are expressed as mean ± standard deviation (SD) in in vitro experiments and mean ± standard error of the mean (SEM) in animal experiments. Statistical significance was determined by one-way analysis of variance (ANOVA) with Dunnett’s post hoc test or two-way ANOVA with Bonferroni’s post hoc test. Results were considered statistically significant when *p* values were less than 0.05.

## Results and Discussion

### WEMA Inhibits Osteoclast Differentiation

**FIGURE 1:**
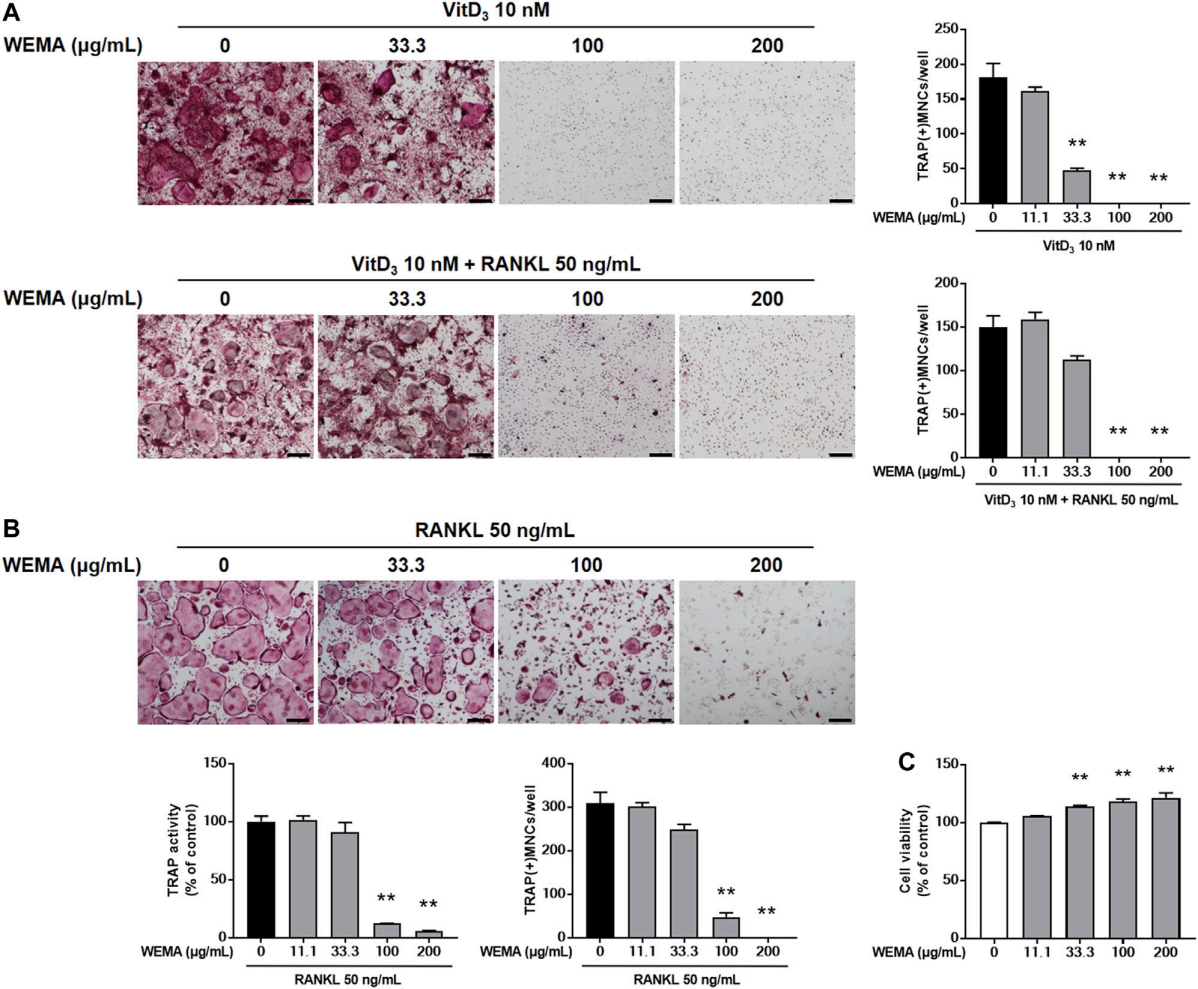
WEMA inhibits osteoclast differentiation. **(A)** MLO-Y4 cells and BMMs were co-cultured with or without WEMA (11.1, 33.3, 100, and 200 μg/ml) for 5 days in the presence of VitD3 (10 nM) or VitD3 (10 nM) plus RANKL (50 ng/ml). Representative images of TRAP staining (left panel; scale bar, 200 µm) and the number of TRAP-positive MNCs containing three or more nuclei (right panel). **(B)** BMMs were cultured with or without WEMA for 4 days in the presence of RANKL (50 ng/ml). Representative images of TRAP staining (upper panel; scale bar, 200 µm), TRAP activity (lower left panel), and the number of TRAP-positive MNCs (lower right panel) containing three or more nuclei. **(C)** BMMs were incubated with or without WEMA (11.1, 33.3, 100, and 200 μg/ml) for 24 h. Cell viability was evaluated by Cell Counting Kit-8 assay. Values are the mean ± SD of one representative experiment out of three with similar results, performed in triplicates. **p < 0.01 versus control.

In remodeling bone, osteocytes are the major cells supporting osteoclast differentiation by providing RANKL (Xiong et al., 2015). MLO-Y4, an osteocyte-like cells, can support osteoclast differentiation in co-culture with osteoclast precursor cells (Zhao et al., 2002). To investigate the effect of WEMA on osteoclastogenesis, we first examined whether WEMA affects osteoclast formation in co-culture of MLO-Y4 with osteoclast precursor cells, BMMs. Treatment of the co-culture with VitD3 for 5 days promoted osteoclast differentiation, which was suppressed by WEMA in a dose-dependent manner (Figure 1A). The addition of exogenous RANKL to the co-culture did not recover the inhibitory effect of WEMA, suggesting the possibility of direct action of WEMA on osteoclast precursors. To investigate whether WEMA directly inhibits osteoclastogenesis in osteoclast precursors, osteoclast differentiation was induced by directly adding exogenous RANKL to osteoclast precursor BMMs. Consistent with the results of the co-culture system, WEMA inhibited RANKL-induced osteoclast differentiation of BMMs in a dose-dependent manner (Figure 1B). WEMA did not reduce cell viability of BMMs, indicating that the inhibitory effect of WEMA was not due to cytotoxicity (Figure 1C).

### WEMA Inhibits RANKL-Induced NFATc1 Expression

**FIGURE 2:**
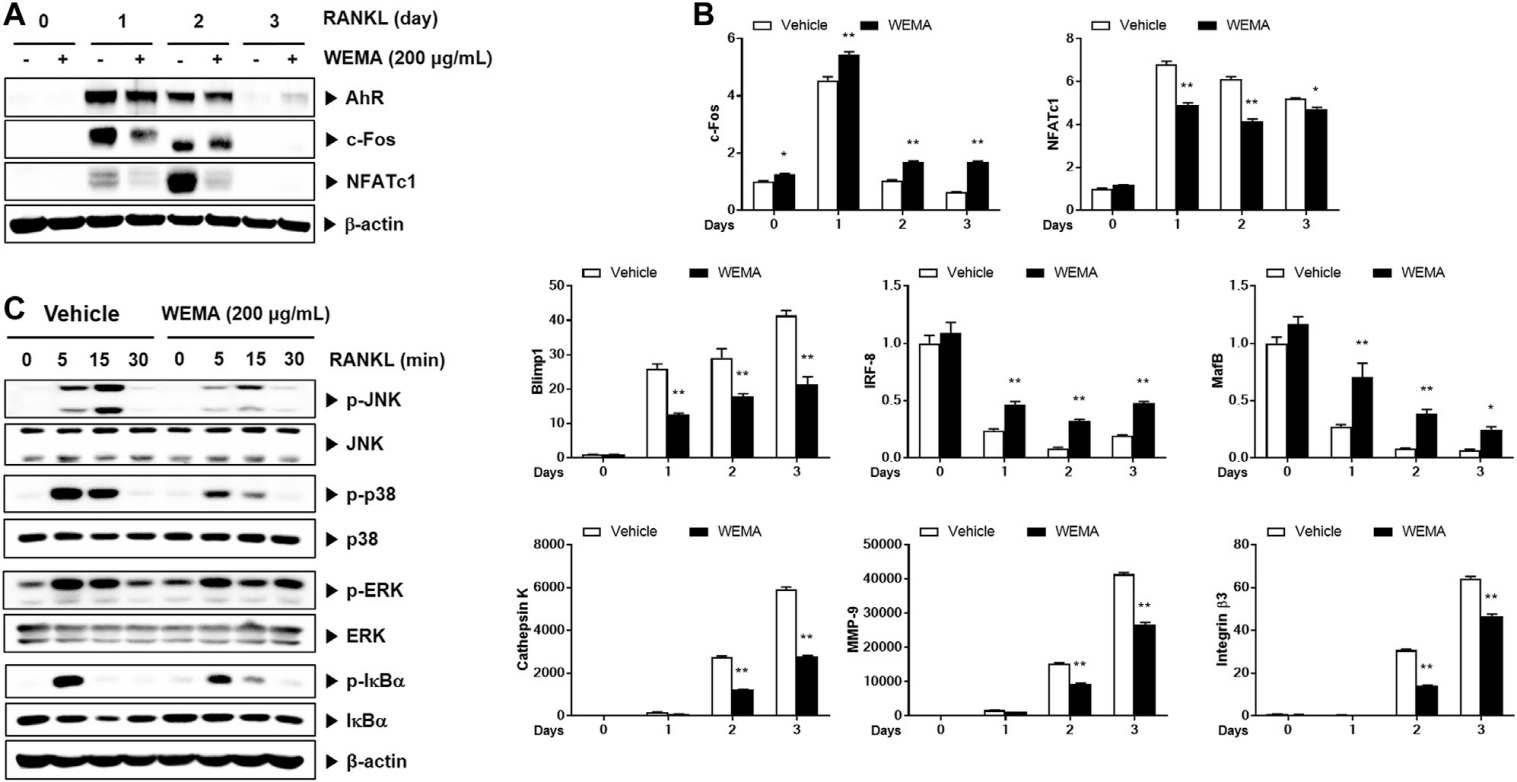
WEMA inhibits RANK-induced signaling required for NFATc1 induction. **(A,B)** BMMs were pre-treated with or without WEMA (200 μg/ml) for 3 h in the presence of M-CSF (60 μg/ml). Then, the cells were stimulated with RANKL (50 ng/ml) for 0, 1, 2, or 3 days. **(A)** The protein expression of AhR, c-Fos, and NFATc1 was analyzed by western blot. **(B)** The mRNA expression of c-Fos, NFATc1, Blimp1, IRF-8, MafB, cathepsin K, MMP-9, and integrin β3 was analyzed by qPCR. **(C)** BMMs were pre-treated with or without WEMA (200 μg/ml) for 3 h in the presence of M-CSF (60 μg/ml). Then the cells were stimulated with RANKL (50 ng/ml) for 0, 5, 15, or 30 min. The indicated proteins were analyzed by western blot. Values are the mean ± SD of one representative experiment out of three with similar results, performed in triplicates. *p < 0.05, **p < 0.01 versus control.

To understand the molecular mechanism underlying the anti-osteoclastogenic effect of WEMA, we investigated its effects on key transcription factors required for osteoclastogenesis. RANKL activates NF-κB and MAPKs in osteoclast precursors, and these early signaling pathways lead to the induction of c-Fos and NFATc1. During osteoclastogenesis, the expression of NFATc1 increases and it functions as a master transcription factor for osteoclastogenesis (Takayanagi et al., 2002). WEMA treatment suppressed RANKL-induced expression of NFATc1 mRNA and protein (Figures 2A,B). Activated NFATc1 is known to regulate osteoclast specific genes such as cathepsin K, MMP-9, and integrin β3 which are important for osteoclast differentiation and bone resorption (Boyle et al., 2003). The WEMA inhibited RANKL-induced mRNA expression of cathepsin K, MMP-9, and integrin β3 (Figure 2B). Recently, it was reported that anti-osteoclastogenic transcription factors, such as IRF-8 and MafB, block the transcriptional activity of NFATc1. These factors are negatively regulated by Blimp-1 (Park et al., 2017). WEMA restored reduced IRF-8 and MafB expression accompanied by enhanced Blimp1 expression during RANKL-induced osteoclastogenesis, indicating that WEMA treatment altered Blimp1 signaling to suppress transcription of NFATc1 and its target genes. c-Fos is also upregulated during osteoclastogenesis and plays a key role in the transcriptional induction of NFATc1 (Matsuo et al., 2004). WEMA inhibited RANKL-induced c-Fos protein expression but not mRNA expression. Recently, AhR, a ligand-activated transcription factor, was shown to mediate RANKL-induced MAPK and NF-κB activation and c-Fos induction (Izawa et al., 2016). WEMA did not affect RANKL-induced AhR induction (Figure 2A). Next, we examined whether WEMA affects RANKL-induced activation of early signaling pathways, including those of MAPKs and NF-κB. It has been reported that p38 is essential in the initial stages of osteoclastogenesis as it promotes the expression of cathepsin K (Matsumoto et al., 2004). JNK is important for osteoclastogenesis, and ERK plays an important role in osteoclast survival (Miyazaki et al., 2000; David et al., 2002). The NF-κB signaling pathway plays a crucial role in the immune and inflammatory response and is a main downstream signaling pathway involved in RANKL-induced osteoclastogenesis (Boyce et al., 2015; Liu et al., 2017). NF-κB exists as an inactive complex with the NF-κB inhibitor IκBα, which prevents nuclear translocation of NF-κB containing the p50 and p65 subunits. However, upon RANKL stimulation, IKK stimulates the phosphorylation and degradation of IκBα and liberates NF-κB, leading to its nuclear translocation (Boyce et al., 2015). WEMA inhibited RANKL-induced JNK and p38 MAPKs phosphorylation, but not ERK MAPK, and it also diminished RANKL-induced NF-κB activation, as determined by phosphorylation and degradation of IκBα (Figure 2C). These results suggest that the anti-osteoclastogenic effect of WEMA is, at least in part, attributable to its inhibition of NFATc1 induction by suppressing RANKL-induced early signaling and/or decreasing c-Fos protein levels.

### WEMA Attenuates OVX-Induced Bone Loss in Mice

**FIGURE 3:**
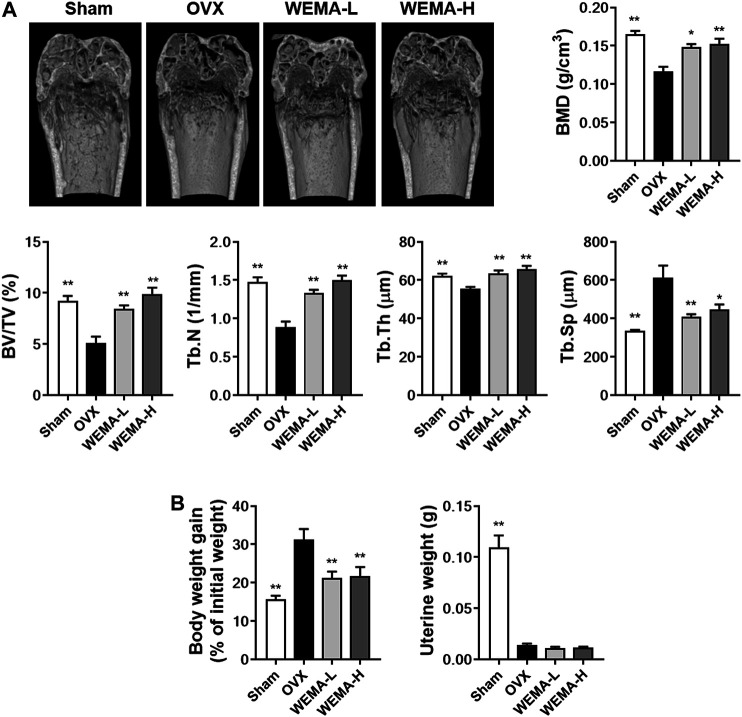
WEMA slowed OVX-induced bone loss in mice. The mice were orally administered vehicle or WEMA for 5 weeks. **(A)** µ-CT images, BMD, and bone morphometric parameters of femoral trabecular bone. **(B)** Body weight gain and uterine weight. OVX, ovariectomy group; WEMA-L, OVX + WEMA 100 mg/kg/day treatment group; WEMA-H, OVX + WEMA 300 mg/kg/day treatment group; BMD, bone mineral density; BV/TV, bone volume per tissue volume; Tb.N, trabecular number; Tb.Th, trabecular thickness. Tb. Sp, trabecular separation; *p < 0.05, **p < 0.01 versus the OVX group.

Having established that WEMA inhibits osteoclast differentiation, we next examined the effect of WEMA on osteoclast-mediated bone loss in OVX mice. OVX is the most common animal model used to evaluate the mechanisms of postmenopausal osteoporosis and therapeutic strategies for treating this disease. Estrogen deficiency by OVX induces osteoclast differentiation at a rate higher than that of bone formation, which accelerates the turnover rate of bone remodeling, resulting in a decrease in bone mass and deterioration in bone architecture (Komori, 2015). OVX in mice is known to significantly affect trabecular bone but not cortical bone (Komori, 2015). To investigate the effect of WEMA on OVX-induced bone loss, WEMA was orally administered for 5 weeks, starting 1 week after OVX. A new high resolution digital imaging technique µ-CT was used to evaluate trabecular bone quantity and quality by measuring BMD and microstructural parameters including BV/TV, Tb.N, Tb.Th, and Tb.Sp in the distal femurs. Compared with the sham group, OVX mice exhibited a marked trabecular bone loss with a decrease in BMD, BV/TV, Tb.N, and Tb.Th and an increase in Tb. Sp, which was remarkably attenuated by WEMA administration (Figure 3A). As expected, estrogen deficiency by OVX resulted in increased body weight gain and uterine atrophy, and WEMA inhibited OVX-induced increase in body weight gain, but not uterine atrophy (Figure 3B). However, we cannot completely exclude the possible involvement of an estrogen-like activity in exerting the anti-osteoporotic and anti-obesity effects of WEMA, given that phytoestrogens can modulate estrogen receptors (ERs) in a tissue-dependent fashion, due to their differential binding affinities to two ER isoforms, ERα and ERβ (Anandhi Senthilkumar et al., 2018). Taken together, our findings indicate that WEMA can attenuate OVX-induced bone loss and weight gain without exerting an estrogenic effect in the uterus, suggesting that WEMA might be an excellent candidate for the prevention or treatment of postmenopausal osteoporosis and obesity.

### Phytochemical Profiles of WEMA

**FIGURE 4:**
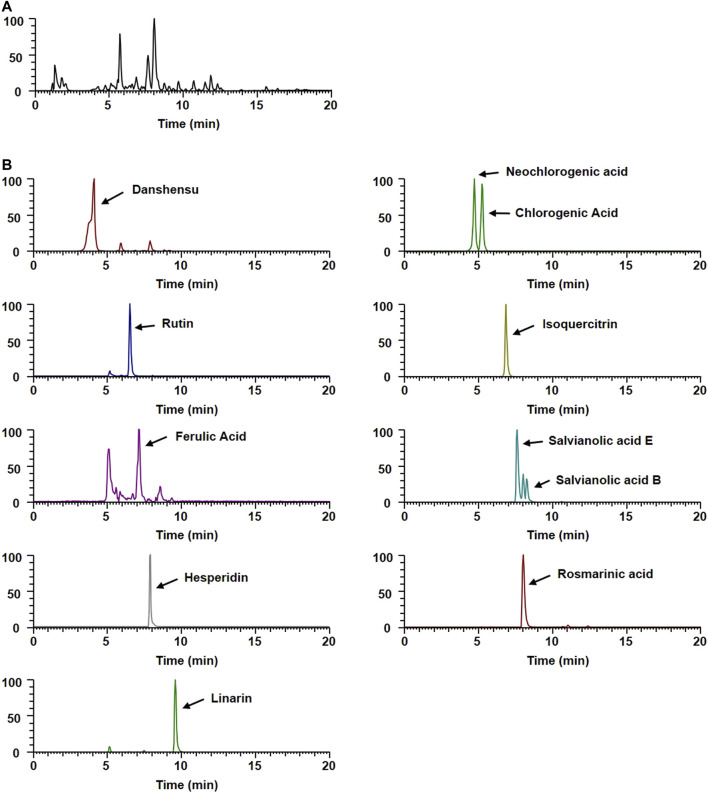
UHPLC–MS/MS analysis of WEMA. **(A)** Base peak chromatogram of WEMA. **(B)** Extracted ion chromatograms of identified phytochemicals.

To gain insight into the potential underlying mechanisms and biological properties of WEMA, we investigated the phytochemical profiles of WEMA. Previous reports have shown that water extracts of *Mentha* species including *M. arvensis* mainly comprise phenolic acids and flavonoids (Koşar et al., 2004; Fecka and Turek, 2007). In the present study, the UHPLC–MS/MS analysis of WEMA identified eight phenolics (danshensu, neochlorogenic acid, chlorogenic acid, rutin, ferulic acid, salvianolic acid E, rosmarinic acid, and salvianolic acid B) and three flavonoids (isoquercitrin, hesperidin, and linarin) (Table 1). Most phytochemicals were determined by comparing with the retention times and mass fragmentations, and salvianolic acid B and E were tentatively identified according to a previous report (Hanafy et al., 2017). The typical UV base peak chromatograms and the extracted ion chromatograms for each WEMA analyte are shown in Figure 4. These phytochemicals, except neochlorogenic acid and salvianolic acid E, have been reported to show bone protective properties *in vitro* and/or *in vivo*. Chlorogenic acid, ferulic acid, and linarin have been shown to inhibit osteoclast differentiation *in vitro* by interfering with RANKL-induced NF-κB activation and NFATc1 induction (Kwak et al., 2013; Omori et al., 2015; Doss et al., 2018; Wang et al., 2018). Danshensu, rutin, isoquercitrin, ferulic acid, hesperidin, rosmarinic acid, and salvianolic acid B were shown to attenuate OVX or RANKL-induced bone loss *in vivo* (Horcajada-Molteni et al., 2000; Chiba et al., 2003; Sassa et al., 2003; Lee et al., 2015; Qu et al., 2016; Liu and Shen, 2018; Fayed et al., 2019). In addition, danshensu, rutin, isoquercitrin, ferulic acid, hesperidin, rosmarinic acid, salvianolic acid B, and linarin were observed to promote osteoblast differentiation (Huan-qiong and Liao, 2008; Trzeciakiewicz et al., 2010; Li et al., 2016; Du et al., 2017; Zhang et al., 2017; Li et al., 2019; Jeong et al., 2021; Liu et al., 2021). Thus, these findings suggest that the beneficial effects of these phytochemicals may account for the anti-osteoclastogenic and anti-osteoporotic effects of WEMA.

**TABLE 1:** Phytochemicals of WEMA by UHPLC-MS/MS.

No	R_t_ (Min)	Calculated (m/z)	Estimated (m/z)	Adducts	Error (ppm)	Elemental composition	MS/MS fragments (m/z)	Identifications
1	4.22	197.046	197.045	[M-H]^-^	−4.43	C_9_H_10_O_5_	197, 179, 135	Danshensu^a^
2	4.69	353.088	353.088	[M-H]^-^	−0.46	C_16_H_18_O_9_	191, 179, 135	Neochlorogenic acid^a^
3	5.21	353.088	353.088	[M-H]^-^	−0.46	C_16_H_18_O_9_	191, 179, 173	Chlorogenic acid^a^
4	6.51	609.146	609.147	[M-H]^-^	0.61	C_27_H_30_O_16_	301, 300	Rutin^a^
5	6.78	463.088	463.088	[M-H]^-^	0.25	C_21_H_20_O_12_	463, 300, 301	Isoquercitrin^a^
6	7.07	193.051	193.050	[M-H]^-^	−3.80	C_10_H_10_O_4_	178, 149, 134	Ferulic acid^a^
7	7.58	717.146	717.147	[M-H]^-^	1.63	C_36_H_30_O_16_	339, 321, 295	Salvianolic acid E
8	7.9	609.183	609.183	[M-H]^-^	0.11	C_28_H_34_O_15_	301	Hesperidin^a^
9	7.99	359.077	359.077	[M-H]^-^	−0.51	C_18_H_16_O_8_	197, 179, 161	Rosmarinic acid^a^
10	8.22	717.146	717.146	[M-H]^-^	1.71	C_36_H_30_O_16_	339, 331	Salvianolic acid B
11	9.51	637.177	637.178	[M + HCO_2_]^-^	0.22	C_28_H_32_O_14_	283	Linarin^a^

a Compared with the retention time (Rt) and mass spectral data of reference standards.

## Conclusion

This study is the first to show the beneficial effects of WEMA on bone health. WEMA inhibited osteoclast differentiation by suppressing RANK signaling to NFATc1 induction in osteoclast precursor cells. In a postmenopausal mouse model, WEMA exhibited beneficial effects on bone loss and weight gain without promoting uterine hypertrophy. In addition, we detected phytochemicals in WEMA that have anti-osteoclastogenic or anti-osteoporotic properties. Collectively, these results suggest that WEMA is a promising herbal candidate that can be used to prevent or treat postmenopausal osteoporosis.

## Data Availability

The original contributions presented in the study are included in the article/supplementary material, further inquiries can be directed to the corresponding author.
